# Natural and Synthetic PPARγ Ligands in Tumor Microenvironment: A New Potential Strategy against Breast Cancer

**DOI:** 10.3390/ijms21249721

**Published:** 2020-12-19

**Authors:** Giuseppina Augimeri, Luca Gelsomino, Pierluigi Plastina, Cinzia Giordano, Ines Barone, Stefania Catalano, Sebastiano Andò, Daniela Bonofiglio

**Affiliations:** 1 Department of Pharmacy, Health and Nutritional Sciences, University of Calabria, 87036 Arcavacata di Rende (CS), Italy; giusy.augimeri@gmail.com (G.A.); luca.gelsomino@unical.it (L.G.); pierluigi.plastina@unical.it (P.P.); cinzia.giordano@unical.it (C.G.); ines.barone@unical.it (I.B.); stefcatalano@libero.it (S.C.); sebastiano.ando@unical.it (S.A.); 2 Department of Pathology, University of Michigan Medical School, Ann Arbor, MI 48109, USA; 3 Centro Sanitario, University of Calabria, 87036 Arcavacata di Rende (CS), Italy

**Keywords:** peroxisome proliferator-activated receptor gamma, PPARγ ligands, breast cancer, tumor microenvironment, cancer-associated adipocytes, cancer-associated fibroblasts, tumor-associated macrophages, tumor endothelial cells, extracellular matrix components, extracellular vesicles

## Abstract

Multiple lines of evidence indicate that activation of the peroxisome proliferator-activated receptor γ (PPARγ) by natural or synthetic ligands exerts tumor suppressive effects in different types of cancer, including breast carcinoma. Over the past decades a new picture of breast cancer as a complex disease consisting of neoplastic epithelial cells and surrounding stroma named the tumor microenvironment (TME) has emerged. Indeed, TME is now recognized as a pivotal element for breast cancer development and progression. Novel strategies targeting both epithelial and stromal components are under development or undergoing clinical trials. In this context, the aim of the present review is to summarize PPARγ activity in breast TME focusing on the role of this receptor on both epithelial/stromal cells and extracellular matrix components of the breast cancer microenvironment. The information provided from the in vitro and in vivo research indicates PPARγ ligands as potential agents with regards to the battle against breast cancer.

## 1. Introduction

Breast carcinoma is the most frequent cancer and cause of cancer-related death in women worldwide, with approximately 2 million new estimated cases and 627.000 deaths in 2018 [1]. Despite the ongoing efforts to improve breast tumor management, early diagnosed breast cancer is considered a curable disease in only 70–80% of patients, whereas breast metastatic carcinoma, accounting 10% of all diagnosed cases, is still an incurable disease for which the major goal of the therapy is to prolong survival and to maintain the quality of life. To date, the clinical treatment plan for breast cancer patients is based on the histological and molecular profile of the tumor. However, patients within the same tumor subtype frequently show different responses to radiotherapy, chemotherapy and targeted therapies, suggesting that new insights are needed as a step toward precision medicine [2]. Over the last few decades, it has been reported that breast cancer consists not only of epithelial cancer cells, but also of the surrounding tumor microenvironment (TME), composed by adipocytes, fibroblasts, immune cells, endothelial cells, pericytes, mesenchymal cells, extracellular matrix, soluble molecules and extracellular vesicles [3]. Interestingly, it has been demonstrated that a dynamic interaction existing between breast cancer cells and the other components of the TME impacts breast tumor progression influencing the effectiveness of the therapeutic treatment [4,5]. Thus, targeting both the epithelial cells and the components of the TME has emerged as a new challenge to provide a better outcome for breast cancer patients [4]. Remarkably, it has been demonstrated that different proteins are co-expressed in the epithelium and in the stromal compartment of breast cancer, representing promising targets for integrative approaches in breast cancer treatment [5]. Over the last few years, peroxisome proliferator-activated receptor γ (PPARγ), a nuclear receptor involved in adipogenesis, has received much attention in breast cancer tumorigenesis for its ability to exert anti-tumor effects through a dual action in breast cancer cells as well as in the components of the TME. PPARγ binding to ligands after its heterodimerization with retinoid X receptor (RXR) regulates the expression of multiple target genes by binding to DNA sequence elements, termed PPAR response elements (PPREs). Different studies have reported that activation of PPARγ by natural or synthetic ligands, such as omega (ω)-3 polyunsaturated fatty acids (PUFAs) and thiazolidinediones (TZDs), respectively, reduces breast cancer cell growth, migration and invasion in different breast cancer cell lines [6,7,8]. Moreover, activation of PPARγ in several cells of the TME, including macrophages and fibroblasts, induces a shift towards less aggressive phenotypes, thus negatively impacting breast cancer progression [7,9]. Here, we will review the role of ligand-activated PPARγ on the epithelial/stromal cells (cellular part) and extracellular matrix (ECM) components/extracellular vesicles (non-cellular part) of the breast cancer microenvironment, highlighting its potential as a novel therapeutic strategy targeting directly cancer cells and/or indirectly disrupting cellular interaction within TME which sustains breast cancer progression.

## 2. Search Strategy and Data Extraction

The literature search for relevant articles using the terms “PPARγ” AND “breast cancer” was performed in Pubmed database. A total of 472 results was found between 1997 and 2020. When we used as keywords “PPARγ” and “tumor microenvironment”, only 104 papers were published from 2002 to 2020 which were restricted to 19 results with the terms “PPARγ” and “breast tumor microenvironment” from 2008 to 2020. In order to focus our attention on different components of TME, we also used the following key words: “PPARγ” and “cancer-associated adipocytes”, “cancer-associated fibroblasts”; “tumor-associated macrophages”; “tumor endothelial cells”; “extracellular matrix”, “extracellular vesicles”, “exosomes”. All articles selected were full-text articles written in English. We also identified further relevant articles from the reference lists of selected papers. The data reviewed have been organized in separate sections focusing on the functional role of PPARγ in the breast TME, including: (i) epithelial cells; (ii) different stromal components; (iii) extracellular matrix components and extracellular vesicles as non-cellular part of TME. Finally, PPARγ ligands have been described as a potential therapeutic tools in the breast cancer microenvironment.

## 3. Functional Role of Proliferator-Activated Receptor γ (PPARγ) in Breast Tumor Epithelial Cells

### 3.1. Epithelial Breast Cancer Cells

Several studies have been conducted to provide a global view of the functional role of PPARγ in breast cancer since it has been demonstrated that it is over-expressed in several breast cancer cell lines. Although both luminal estrogen receptor (ER)α-positive (MCF-7 and T47D cells) and claudin-low triple negative breast cancer (TNBC) (MDA-MB-231 and BT549 cells) cell lines express PPARγ, higher protein levels of this receptor were found in the TNBC cell lines than in the luminal subtype lines [10], suggesting that PPARγ may represent a molecular target for treatment of the more aggressive breast cancer phenotype. Indeed, ligand-activated PPARγ induces breast cancer cell differentiation into a less malignant phenotype, enhancing the expression of markers of normal breast development [11]. It is noteworthy that PPARγ, as a part of the endocannabinoid signaling system [12], is a molecular target of the main endocannabinoid compound, anandamide. Although most studies have focused on other forms of cancer and not breast cancer in itself [13], potential anticancer effects were observed on breast cancer cell lines following treatment with URB597, a known inhibitor of endocannabinoid catabolism. The fact that these effects were not linked to either CB1, CB2 or TRPV1 activation could suggest a role for PPARγ [14]. Natural and synthetic PPARγ ligands reduce tumor growth through several molecular mechanisms, including cell cycle arrest, autophagy and apoptosis. In particular, PPARγ ligands reduce protein levels of different cyclin-dependent kinases (CDKs), including CDK4 and CDK2, and their regulatory subunits, such as cyclin D1, inducing G0-G1 cell cycle arrest [15,16]. Similarly, ligand-activated PPARγ also promotes G0-G1 cell-cycle arrest in breast cancer cells enhancing the expression of the tumor suppressor p53 and its effector p21. Interestingly, authors demonstrated that the synthetic and specific PPARγ ligand rosiglitazone increases p53 gene promoter transcriptional activity through direct recruitment of PPARγ to the NF-κB sequence localized within the p53 promoter region in a PPARγ-dependent manner [17]. In addition, it has been reported that ligand-activated PPARγ regulates several signaling pathways involved in tumorigenesis as well as other molecular mechanisms to inhibit breast cancer proliferation. Indeed, it has been demonstrated that the anti-proliferative effects of PPARγ in MCF-7 breast cancer cells are mediated, at least in part, by the opposite interplay exerted by ERα and PPARγ pathways on the phosphatidylinositol 3-kinase (PI3K)/protein kinase B (AKT) signaling, whose activation induces breast cancer cell proliferation. Rosiglitazone induces a dose-dependent negative interference with the PI3K/AKT cascade through the upregulation of tumor suppressor gene phosphatase and tensin homolog on chromosome ten (PTEN), thus determining breast cancer cell growth inhibition in MCF-7 cells [18]. Moreover, it has been reported that ligand-activated PPARγ can also reduce tumor growth counteracting the leptin-signaling [19], which is well-known to sustain breast cancer progression [20]. Indeed, rosiglitazone was revealed to prevent leptin-induced tumor growth in nude mice and to inhibit proliferation in breast cancer cells upon leptin treatment [19]. Notably, natural and synthetic PPARγ ligands activate different cell death programs as another modality to cause cell growth suppression. In particular, ligand-activated PPARγ triggers autophagy in breast cancer cells regulating the expression of key molecules involved in this process. It has been reported that sub-saturated doses of the TZDs, troglitazone and rosiglitazone, activate the autophagic flux in a PPARγ-dependent manner through the upregulation of the hypoxia-inducible factor 1 (HIF1)α in MDA-MB-231 breast cancer cells [21]. Moreover, several ω-3 PUFA conjugates, including dopamine and ethanolamine derivatives, were shown to induce autophagy increasing the expression of Beclin-1 in a transcriptional and non-transcriptional manner [8,22]. Besides the activation of the autophagic flux, ligand-activated PPARγ exerts also pro-apoptotic effects in breast cancer cells, thus inducing cell growth suppression. The natural PPARγ agonist 15-deoxy-Δ12,14-prostaglandin J2 (15d-PGJ2) increases the production of reactive oxygen species (ROS) and reduces the O_2_ consumption in the mitochondria, inducing intrinsic apoptosis [23], whereas the synthetic PPARγ ligand rosiglitazone was evidenced to stimulate the FAS/FAS ligand signaling pathway and the sub-sequential cleavage of the caspase-8, resulting in the activation of the extrinsic apoptosis [24]. 6-Shogaol, the major bioactive compound in the rhizomes of ginger, generates growth inhibition and apoptosis in a PPARγ-dependent manner suppressing NF-κB activity in breast cancer cells [25]. Moreover, the ω-3 PUFA docosahexaenoic acid (DHA) acting as an agonist of PPARγ stimulates apoptosis in breast cancer cells increasing the expression of the tumor suppressor syndecan-1 (SDC-1) [26]. Interestingly, the conjugates of eicosapentaenoic acid (EPA) and DHA with dopamine, named EPADA and DHADA, respectively, trigger both autophagy and apoptosis in a time-dependent manner [8]. Accumulating evidence reveals that autophagy which is a classically cytoprotective mechanism, may precede apoptosis in the complex interplay between these cell death processes governing cell fate [27]. Indeed, after 24 h of treatment, EPADA and DHADA increase beclin-1 transcriptional activity in breast cancer cells, activating the autophagic flux. However, for long-term exposure, DHADA and EPADA block autophagy inducing the cleavage of caspase-9 and beclin-1 and activate the apoptotic cascade, enhancing the cytochrome c release into the cytoplasm and the DNA fragmentation in different breast cancer cell lines [8]. Furthermore, the combination of PPARγ ligands with other drugs potentiates the pro-apoptotic effects exerted by activation of PPARγ in breast cancer, suggesting a promising role of this receptor in the multidrug approach therapy [28]. Michael et al. demonstrated a synergistic growth inhibition and apoptosis in MDA-MB-231 breast cancer cells upon combined treatment with the TZD ciglitazone and the COX-2 inhibitor NS-398 [29]. In addition, our research group demonstrated that the combination of rosiglitazone with the RXR ligand 9-*cis*-retinoic acid induces apoptosis in different breast cancer cell lines through the activation of the intrinsic apoptosis. Indeed, the combination of these compounds induces the release of the cytochrome c from the mitochondria to the cytoplasm and the cleavage of caspase-9, in a p53-dependent manner [30]. However, combined treatment of rosiglitazone with 9-*cis*-retinoic acid can also trigger the intrinsic pathway in a p53-independent manner though the upregulation of the pro-apoptotic Bid and the formation of a p53-Bid complex [31]. Moreover, using triple immune-deficient BNX mice Elstner et al. have demonstrated that troglitazone alone or in combination with the RXR agonist all-*trans*-retinoic acid (ATRA), inhibits tumor growth and apoptosis in vivo, reducing tumor size and tumor weight [28]. Further investigations have demonstrated that activation of PPARγ can counteract breast cancer cell invasion and migration. In particular, Rovito et al. have revealed that rosiglitazone negatively regulates C-X-C chemokine receptor type 4 (CXCR4) expression through the recruitment of the silencing mediator of retinoid and thyroid hormone receptor (SMRT) co-repressor onto a PPRE localized within CXCR4 promoter region, thus reducing breast cancer cell invasion and migration [7]. The anti-tumoral effects of PPARγ activation is summarized in Table 1.

### 3.2. Breast Cancer Stem Cells

Breast cancer stem cells (BCSCs), which are characterized by their ability to undergo self-renewal and to differentiate into the non-self-renewing cells forming the tumor bulk, have been shown to drive breast tumor growth and recurrence [32,33]. Therefore, BCSC-related therapeutic options may be a valid strategy for the treatment of breast cancer, especially in the case of therapeutic resistance. In this context, contrasting data are reported on the role of PPARγ as tumor suppressor, since it has been reported that antagonizing PPARγ signaling decreases cancer stem cell population in Erb-B2 receptor tyrosine kinase 2/human epidermal growth factor receptor 2 (ERBB2/HER2)-positive human breast cancer and inhibits tumor formation in an animal model [34], whereas PPARγ downregulation has been associated with Wnt/β-catenin upregulation that is a crucial regulator of stem cells, stem progenitors and cell self-renewal [35,36]. Mechanistically, the canonical Wnt/β-catenin pathway and PPARγ signaling work in an opposite manner in cancers generally creating a vicious circle. Indeed, PPARγ negatively affects the c-Myc/Wnt/β-catenin axis and stimulates β-catenin proteasome degradation, while activation of Wnt signaling induces inactivation of PPARγ [36]. In addition, the Wnt/β-catenin pathway exerts a positive regulation on pro-inflammatory cytokines and oxidative stress which in turn stimulates BCSCs to drive tumor initiation and progression [37]. Conversely, the combination of PPARγ and RXR ligands reduces the expression and activity of HIF1α and blunts the pro-inflammatory phenotype of BCSCs, underlining a link between hypoxia and inflammatory pathways in sustaining BCSCs [38]. Notably, HIF1α can activate the Wnt pathway, stimulating the self-renewal of BCSCs through a direct activation of cancer stemness capability, but also inhibiting PPARγ activity [39,40]. Accordingly, by investigating the interaction between PPARγ and the Wnt pathway in human breast carcinoma, elevated levels of PPARγ and a low amount of β-catenin in para-cancerous respect to breast cancer tissues have been detected, indicating an inverse correlation between PPARγ and β-catenin expression [41]. Recently, in breast cancer tissues the expression levels of PPARγ were investigated in relation to many clinicopathological parameters including patient survival. Interestingly, only cytoplasmic receptor had a strong correlation with poor survival and was associated with high-risk markers of breast cancer such as HER2, the cancer cell marker CD133 and N-cadherin, a well-known indicator for epithelial-to mesenchymal transition (EMT), while nuclear PPARγ expression was negatively correlated with tumor grade as well as with HER2 and N-cadherin expression [42]. On the other hand, Jiang et al. have found that high expression levels of PPARγ were associated with long patients’ overall survival, suggesting the clinical relevance of this receptor as a prognostic indicator potentially targetable for the development of novel treatments in breast cancer [41]. Based on the genomic action of the receptors, which requires specific compartmentalization, it is reasonable to hypothesize that nuclear activation of PPARγ acting as tumor suppressor exerts a potential protective role against breast cancer development, whereas the presence of inactive cytoplasmic PPARγ could be a marker of poor prognosis in breast cancer patients.

## 4. Functional Role of PPARγ in the Stromal Cell Components of Breast Tumor Microenvironment

Breast cancer epithelial cells develop in a complex and dynamic stromal microenvironment that influences cell growth, invasion, and metastasis. The interaction of breast cancer cells with their microenvironment is bidirectional and includes: (i) cell–cell contacts, involving cancer-associated adipocytes (CAAs), cancer-associated fibroblasts (CAFs), tumor-associated macrophages (TAMs), tumor endothelial cells; (ii) cell-free contacts involving ECM components and the mediators that, as secreted soluble molecules/factors or extracellular vesicles, enable these interactions through the horizontal transfer of signalling and/or genetic information within TME.

### 4.1. Cancer-Associated Adipocytes

In the local breast microenvironment, cross-talk between epithelial cells and adipocytes, which represents a relatively abundant component of breast parenchyma, is essential for the normal development and differentiation of the mammary gland during puberty and maintains ductal architecture and structure in adulthood [43]. In this context, PPARγ is considered the master regulator of adipogenesis since it participates in the transcriptional activation of several adipogenic and lipogenic genes [44,45], also regulating the synthesis of fatty acids via the modulation of mitochondrial citrate carrier expression [46], which represents a crucial cross-point for several metabolic pathways. Adipose tissue is not only a metabolic tissue, but also an endocrine organ able to secrete adipokines, chemokines and cytokines, that play a crucial role in maintaining homeostasis of metabolism and immunity in adipose tissue [47]. In physiological condition, stroma maintains epithelial polarity, inhibits uncontrolled cell growth and neoplastic transformation, however this particular dialog persists also in pathological conditions, such as in cancer [48]. Tumor cells exert substantial effects on adjacent adipocytes resulting in a dedifferentiation process of mature adipocytes which become fewer, lose lipids and acquire fibroblast-like features with increased expression of the fibroblast-specific protein-1 (FSP-1) but not α smooth muscle actin (SMA) [49,50,51]. Adipocytes modified by tumor cells are named cancer-associated adipocytes (CAAs), which differ from the normal adipocytes also in the expression of differentiation markers such as PPARγ and C/EBPα as well as their downstream genes such as fatty acid binding protein 4 (FABP4) and hormone sensitive lipase (HSL) [49]. Reciprocally, the transient interaction between human breast cancer cells and human adipocytes enhances the malignant behavior of the breast cancer cells in vitro and in vivo [52]. In particular, the co-cultured adipocytes which lost the classical terminal differentiation marker PPARγ exhibited a significant decrease in lipid accumulation, and this occurs dramatically in the most aggressive human breast cancer line MDA-MB-231 compared to human MCF-7 breast cancer line with low metastatic potential [52]. Moreover, CAAs have been demonstrated to be involved in tumor progression, metastasis and therapy resistance by secretion of adipokines, such as leptin and a series of inflammatory chemokines and interleukins [51,53], including tumor necrosis factor (TNF) α, Interleukin (IL)1β, IL6, IL8 and vascular endothelial growth factor (VEGF) which are well known for their effects in promoting tumorigenesis [54,55,56,57]. Conversely, adiponectin, whose expression is regulated by PPARγ activated transcriptional program, is decreased in both in vitro co-culture models and in CAAs of human breast cancer tissues as compared with normal mammary adipose tissue [50]. Adiponectin participates in the interaction between tumor cells and adipocytes playing an anti-tumorigenic role by inducing apoptosis, suppressing growth and invasion of breast cancer cells through AMPK activation, PI3K/AKT inhibition and modulation of cyclin D1 levels [58]. Growing in the presence of adipocytes, cancer cells are able to adapt their metabolism taking advantage of metabolites (lactate, ketones, fatty acids) produced by microenvironmental host cells (reverse Warburg effect) [59]. Indeed, fatty acids derived from lipolysis are released by CAAs and utilized by cancer cells to obtain energy from mitochondrial β-oxidation to promote uncontrolled cancer cell growth and tumor progression [60]. This effect represents a metabolic switch from the aerobic glycolisis classically occurring in cancer cells (Warburg effect) which is primarily regulated by HIF-1 signaling. In this cellular context, hypoxia regulates PPARγ activity that, as a key mediator of energy metabolism, impairs glycolytic pathways [40]. Recently, it has been reported that the well characterized miR-155, an oncomiR secreted by cancer cells, alters the metabolism of surrounding adipocytes by downregulating PPARγ expression, accelerating the cancer-lipolytic process associated with tumor progression [61]. The crucial role of PPARγ emerges from the ability of this activated receptor to mediate the energy metabolism of adipocytes suggesting that the regulation of adipocyte energy stores which are sensitive to PPARγ could reveal new anti-tumor therapeutic possibilities [62]. In addition to the deepening of the role of adipocytes in supporting breast cancer progression, over the last few decades much attention has been focused on the link between adipocytes and inflammation within breast cancer. More specifically, the adipose tissue microenvironment hosts inflammatory M1 macrophages which encircling the adipocytes to form the crown-like structures (CLS). The outcomes of this interaction include the activation of M1 macrophages that synergistically contribute to increase lipolysis and reduce triacylglycerol synthesis in the adipocytes resulting in elevated free fatty acid levels which negatively affect metabolic homeostasis [63]. Notably, in breast cancer patients the presence of CLS accumulated in adipose tissue is associated with worse prognosis [64]. However, lipolysis is also essential for the activation of anabolic metabolism and expression of key genes that mark commitment to M2 macrophage polarization [65]. Moreover, PPARγ has been also found to be crucial for the activity of adipose tissue associated-Treg cells, which express this receptor at higher level than Treg originating from lymphoid organs [66]. Activation of PPARγ has been associated with a cluster of mRNAs involved mainly in fatty acid transport, biosynthesis and oxidation, while genetic deletion of PPARγ in vivo led to a contraction of the Treg population in adipose tissue with a relative increase in pro-inflammatory M1 macrophages [67]. To conclude, adipocytes participate in a highly complex vicious cycle orchestrated by cancer cells that reprogram adipocytes which in turn sustain tumor progression. Therefore, disrupting the symbiosis between breast cancer cells and adipocytes should reveal new therapeutic opportunities.

### 4.2. Cancer-Associated Fibroblasts

Fibroblasts are the most abundant stromal cell type population in the mammary gland and represent important players in the normal mammary development as well as in breast cancer tumorigenesis. In the physiological condition, fibroblasts support the deposition of ECM, modulating the mammary epithelium architecture [68], and suppress the hyperplastic growth generated by abnormal epithelial breast cells, thus reducing breast tumor initiation [68,69]. However, once the tumor is established, normal fibroblasts acquire morphological and phenotypical changes that lead to the CAF phenotype, supporting breast tumorigenesis [70]. Indeed, CAFs increase proliferation, migration and invasion of breast cancer cells, enhancing angiogenesis, immunosuppression and metastasis [71]. Thus, CAF-derived signals can affect breast cancer cell phenotype in a paracrine fashion, supporting tumor progression.

Interestingly, it has been reported that ligand-activated PPARγ can inhibit CAF-induced effects on breast cancer cell interfering with the CXCR4/stromal cell-derived factor-1 (SDF-1) α axis, which plays a crucial role in mediating breast cancer cell invasion and metastasis. In particular, it has been demonstrated that rosiglitazone reduces the binding of the SDF 1-α secreted by CAFs to the CXCR4 expressed in MCF-7 and MDA-MB-231 breast cancer cells, thus reducing breast cancer cell motility [7]. To date, the biological consequence of PPARγ activation in CAFs is still controversial. Indeed, Avena et al. have demonstrated that activation of PPARγ in fibroblasts results in a catabolic pro-inflammatory microenvironment that support breast cancer proliferation [72]. Mainly, they revealed that fibroblasts overexpressing PPARγ become autophagic, senescent and glycolytic and produce metabolites which are used by breast cancer cells to increase their mitochondrial capacity and their proliferative ability. In contrast, Rovito et al. showed that activation of PPARγ in CAFs leads to a less aggressive phenotype, characterized by a lower expression of vimentin and α-SMA. Moreover, rosiglitazone treatment was shown to reduce CAF motility, counteracting breast cancer progression [7]. Accordingly, pioglitazone reduced the expression of IL6/IL8 and the promoter activity of NK-kB and IL6 in CAFs, indicating that activation of PPARγ hinders the pro-inflammatory phenotype of CAFs [38].

### 4.3. Tumor-Associated Macrophages

Emerging evidence is in support of a functional association between chronic inflammation and cancer. Recruitment and infiltration of immune cells including macrophages is observed in many tumors [73]. Tumor-associated macrophages (TAMs) are abundant within tumors and their presence has been correlated with poorer prognosis in almost all tumors. TAMs support tumor growth and metastasis by promoting cancer cell proliferation, immunosuppression and angiogenesis [74]. Macrophages are plastic cells displaying divergent phenotypes, and their functions including cytokine production may vary in response to various microenvironmental signals [75,76]. In particular, macrophages are classically divided into two divergent sub-populations, as pro-inflammatory M1 or anti-inflammatory M2 phenotypes. M1 macrophages are characterized by the expression of pro-inflammatory cytokines and have the ability to kill and remove tumor cells, according to their physiological role in phagocytosis [77]. On the other hand, M2-polarized macrophages have been characterized by the expression of anti-inflammatory cytokines and proangiogenic factors such as IL-10 and VEGF, respectively [78]. M2 macrophages play a crucial role in the maintenance and development of both primary and metastatic cancers by contributing to vascular supply and to vascular endothelial cell proliferation, basement membrane breakdown and deposition, recruitment of leukocytes and overall immune suppression [64,79]. From a clinical perspective, some differences have been observed and higher amounts of M1 cells within primary tumors are associated with better prognosis, while a shift towards the M2 phenotype is associated with poor outcomes [80,81]. More recent findings regarding tumor development and progression indicated that this Manichaean scheme is an over-simplification and that the picture is more complex. Indeed, TAMs are not necessarily characterized by M1 or M2 phenotypes, as they can behave in-between or off this spectrum [82].

The possible role of PPARγ in tumorigenesis has been controversially discussed [83]. The polarization of macrophages to the M2 phenotype has been partly linked to PPARγ activation [84]. Niu et al. reported that caspase-1 promotes the differentiation of TAMs by cleaving PPARγ at residue Asp64, thereby generating a 41 kDa fragment. This PPARγ fragment translocates into the mitochondria and binds to medium-chain acyl-CoA dehydrogenase (MCAD). This results in an attenuation of MCAD activity and inhibition of fatty acid oxidation, thus leading to the accumulation of lipid droplets and promoting TAM differentiation. Interestingly, administration of caspase-1 inhibitors or infusion of bone marrow-derived macrophages genetically engineered to overexpress murine MCAD markedly suppresses tumor growth [85]. Shu et al. found that the use of inhibitors of integrin β3, that is highly expressed on the surface of TAMs, both in vivo and in vitro, inhibited M2 polarization of TAMs. Moreover, in a cell model of M2-polarized macrophages, either blockade or knockout/knockdown of integrin β3 could also suppress macrophage M2 polarization, suggesting that the M2 polarization depends on integrin β3. The expression and activation of PPARγ participated in M2 polarization that was mediated by integrin β3. Moreover, treating 4T1 tumor-bearing mice with integrin β3 inhibitors increased M1/M2 ratio of TAMs, while the infiltration of total lymphocytes into tumor tissue was not altered [86]. More studies demonstrate that a number of antineoplastic processes initiated by PPARγ activation in TAMs induce a switch towards a less aggressive phenotype, thus limiting breast cancer progression [9]. The activation of PPARγ with synthetic agonists, such as the TZD anti-diabetic drugs rosiglitazone and pioglitazone, have been implicated to inhibit tumor malignancy. Cheng et al. found that PPARγ inhibits macrophage ability to produce a protein called Gpr132, which in turn sustains inflammation and allows the growth of breast cancer cells [87]. Genetically modified mice in which macrophages could not express the PPARγ protein and thus not produce Grp132 displayed less inflammation, and cancer growth was blocked. Furthermore, breast tumors in the engineered mice did not shrink after treatments with TZDs, whereas tumors of normal mice did [87]. Gionfriddo et al. explored the ability of synthetic and natural PPARγ ligands to modulate TAM polarization generated by adding two different breast cancer cell conditioned media (CM) to the human monocytic THP-1 cells. Resulting macrophages concomitantly exhibited both M1 and M2 phenotypes. Interestingly, synthetic PPARγ agonist rosiglitazone reduced secretion of M1 pro-inflammatory and pro-tumor M2-cytokines. In addition, two ω-3 PUFA conjugates with ethanolamine and serotonin, N-docosahexaenoyl ethanolamine (DHEA) and N-docosahexaenoyl serotonin (DHA-5-HT), respectively, showed a similar inhibitory effect without affecting macrophage polarization. Interestingly, the inhibitory effect of rosiglitazone, DHEA and DHA-5-HT on cytokine secretion by TAMs was reversed by the PPARγ antagonist GW9662, suggesting the potential involvement of PPARγ [9]. The cross-talk between cancer cells and macrophages in metastasis has been investigated also by Kim et al. They demonstrated that the CM from macrophages exposed to apoptotic cancer cells was able to inhibit the transforming growth factor (TGF)β1-induced EMT, migration, and invasion of breast cancer cells. PPARγ activation in macrophages induced the secretion of PTEN in exosomes and the resulting increased levels of exosomal PTEN were taken up by recipient lung cancer cells. A single injection of ApoSQ cells was found to inhibit lung metastasis in mice and enhanced PPARγ/PTEN signaling in TAMs as well as in tumor cells was observed. On the other hand, PPARγ antagonist GW9662 reversed the signaling by PPARγ/PTEN [88]. Huang et al. showed that activation of PPARγ with rosiglitazone in TAMs may induce tumor vessel normalization and reduce TAM infiltration. Additionally, breast tumor bearing mice treated with rosiglitazone in combination with radiotherapy showed a significant reduction in lesion size and lung metastasis [89].

### 4.4. Tumor Endothelial Cells

The endothelium plays a critical role in the growth and spread of cancer [90], since the growth of tumours requires angiogenesis to sustain it. However, tumor endothelial cells also contribute to tumour growth and metastasis through the secretion of proinflammatory transcription factors which in turn regulates cytokine/chemokine and adhesion molecule expressions that are central to inflammatory cell recruitment [91]. The role of PPARγ as a negative regulator of endothelial cell inflammation and angiogenesis has been largely demonstrated [92,93]. Mechanistically, PPARγ can act with multiple actions, such as downregulating VEGF either directly through a PPAR response element located within the VEGF promoter [94] by decreasing VEGF responses through the suppression of transcription of its receptor VEGFR2, by interacting with and preventing Sp1 binding to DNA [95] or by reducing prostaglandin E2, an endogenous stimulator of angiogenesis [96]. In addition, the depletion of PPARγ in endothelial cells impaired angiogenesis through a dysfunctional Wnt/β-catenin signaling and a regulation of gene crucial for endothelial cell homeostasis, suggesting the influence exerted by PPARγ in angiogenic response [97]. Recently, by using an in vivo and in vitro approach and supported by bioinformatic data, the tumor suppressor role of PPARγ has been deciphered in breast cancer [98]. Specifically, PPARγ ligand pioglitazone reduced tumor growth and metastasis in a mouse model as well as inhibiting the VEGF/fibroblast growth factor 2 (FGF2) production and angiogenesis promoted by chronic stress in murine breast cancer cells [98].

## 5. PPARγ in the Non-Cellular Part of the Breast Tumor Microenvironment

### 5.1. Extracellular Matrix Components

The reorganization of ECM, a crucial component of tissues, is fundamental for breast cancer progression, invasion ad metastasis and its deregulation has been recognized as a cancer hallmark [99]. ECM is a highly dynamic complex of structural proteins and includes the interstitial matrix, mainly produced by stromal cells and the basement membrane, known to collaborate in maintaining the structure under epithelial and endothelial cells [100]. Several collagens (I, III, V, VI, VII and XII), proteoglycans and glycoproteins (tenascin-c and fibronectin) have been found in the interstitial matrix, and some of them usually resulted up-regulated in breast cancer [101]. Different enzymes can modify ECM and among these, the matrix metalloproteinases (MMPs) play a pivotal role in breast cancer progression. In particular, tumor cells secrete MMP-2 and MMP-9, enzymes able to degrade collagen type IV, the main protein associated with the basement membrane, in order to invade other tissues [101]. Besides the well-known role of PPARγ ligands in reducing breast tumor cell proliferation, Liu et al. were the first attributing to PPARγ a role in modulating tumor cell invasion. These authors showed that PPARγ ligands reduced the invasive capabilities of MDA-MB-231 breast cancer cells, enhancing the ratio of metallopeptidase inhibitor 1 (TIMP-1), the tissue inhibitor of MMPs, to MMP-9 with a consequent reduction of the activity of this enzyme [102]. In this context, Hwang et al. also reported that DHA modulated MMP-9 expression and thus MCF-7 breast cancer cell invasion. Mainly, the activation of PPARγ induced by DHA led to the inhibition of NF-kB activity with a consequently decreased MMP-9 expression [6]. Furthermore, Hong et al. supported these data by confirming that troglitazone, through NF-kB/AP-1 suppression, blocked MMP-9 expression and reduced MCF-7 cell invasion [103]. In another elegant work, a link between PPARγ and parvin-β, a protein downregulated in breast cancer cells, has been described. This molecule is a focal adhesion protein that inhibits the activity of integrin-linked kinases (ILKs), key players in the interaction between cell surface integrins and the actin-binding proteins. Besides this structural role, ILKs are involved in cancer growth and invasiveness. The re-expression of parvin-β in MDA-MB-231 cells inhibited tumor growth in the xenograft model and concomitantly induced the up-regulation of the PPARγ mRNA levels and its activation [104]. Nowadays, the role of PPARγ in influencing another important component of ECM, the plasminogen activator inhibitor type-1 (PAI-1) is still controversial. PAI-1 is associated with a poor prognosis in breast cancer patients and is involved in the blockade of plasminogen into its active serine protease, plasmin. The serine protease urokinase plasminogen activator (uPA) is able to induce plasmin and this enzyme increases uPA creating a positive feedback loop. Plasmin degrades the ECM directly or through the activation of MMPs. However, as reviewed by Carter and colleagues, it is likely that PPARγ activation may affect PAI-1 expression and might reduce uPA expression leading to a less aggressive cell tumor phenotype in breast tissue through NF-kB downregulation [105]. These data support the idea that ligand-induced PPARγ activation by modulating ECM components may prevent tumor cell spread and metastasis.

### 5.2. Extracellular Vesicles/Exosomes

In recent decades, cancer research has been focused on the role of extracellular vesicles (EVs) which represent an important mode of intercellular communication and a potential innovative target in breast cancer. Among the large family of EVs, exosomes play a pivotal role in cancer cell-to-cell communication and exert pleiotropic functions that influence breast cancer biology significantly, from initiation to tumor dissemination. These EVs are small lipid bilayer particles (30–150 nm) secreted by both normal and malignant cells and are usually found in several bodily fluids (i.e., urine, serum, plasma, breast milk and saliva) [106]. Exosomal cargoes (mainly several biomolecules such as microRNAs, mRNAs, DNAs, proteins and, lipids), through their delivery into recipient cells, are the mediators of the exosome’s effects [107,108]. Interestingly, proteomic analysis revealed the presence of PPARγ as an exosome-associated protein that circulates in human plasma [109]. Nevertheless, the role of PPARγ as a component of exosomal cargo is still under investigation. Recently, one study reported that PPARγ is a direct target of miR-155 that has been found encapsulated in exosomes from breast cancer patients [110]. It has been described how breast cancer cells over-expressing miR-155 exhibited a down-regulation of PPARγ expression and, consequently, decreased lipid droplets in mature adipocytes. Moreover, miR-155 reprogramming the metabolism of adipocytes triggered cancer-associated cachexia, a condition often associated with advanced cancer and metastasis [61]. Papi et al. demonstrated that mammosphere formation induced by exosomes derived from MCF-7 cells was reduced when exosomes were obtained from breast cancer cells treated with PPARγ and RXR agonists [31]. In addition, exosomes may stimulate the activation of fibroblasts within TME, promoting a protumorigenic phenotype while PPARγ/RXR agonists blunt this activity, suggesting the ability of ligand-activated PPARγ to interrupt exosomal signals to surrounding BCSCs [38]. However, the connection between exosomes and PPARγ still represents an unexplored research avenue, and thus investigating the future the role of this receptor as an exosomal cargo or as an exosome specific target may shed new light in cancer prevention and treatment.

## 6. PPARγ Ligands as Potential Therapeutic Tools in the Breast Cancer Microenvironment

Since the TME components are increasingly recognized as crucial players in breast cancer progression, targeting tumor hosts became the new challenge for breast cancer treatment. To date, three different approaches targeting the breast cancer microenvironment, consisting of the aromatase, angiogenesis and HER2 inhibitors, have been approved for the management of breast cancer disease. However, research on the TME target therapy remains still ongoing in order to discover a good strategy to educate the breast TME without disrupting important homeostatic functions. Among the TME components, immune cells have been investigated as a target in breast cancer microenvironment. In particular, it has been proposed that inhibiting macrophage recruitment and differentiation into TAMs or suppressing the chronic inflammation supplied by adaptive immune cells enhances the efficacy of the chemotherapy and improves breast cancer prognosis [111]. Interestingly, ligand-activated PPARγ was shown to attenuate M1 and M2 polarization of breast TAMs, representing good tools to maintain macrophages in an inactive state that does not affect breast cancer progression [9]. Besides the control of the immune systems, different therapeutic options targeting CAFs are currently under investigation. Lysyl oxidase (LOX) and MMP inhibitors were evidenced to regulate the extracellular matrix remodeling, improving the drug delivery efficacy in in vitro and in vivo breast cancer models [112,113]. Natural and synthetic PPARγ ligands demonstrated to counteract the activity of different MMPs in different breast cancer cell lines, suggesting their potential role in enhancing the effectiveness of the standard breast cancer chemotherapeutic agents in a multidrug therapeutic approach [6,102,103]. Another strategy to target CAFs consists in modulating the paracrine signaling between CAFs and breast cancer cells [114]. In this context, the CXCR4 inhibitors, which antagonize the effects of the CXCR4 ligand SDF-1α secreted by CAFs preventing the development of breast cancer cell metastasis, have received much interest [115]. The encouraging data supporting the ability of ligand-activated PPARγ in blocking the SDF-1α/CXCR4 axis in breast cancer in vitro models represent a good starting point for further clinical studies investigating the possible action of PPARγ ligands in reducing breast cancer metastasis though the regulation of this pathway [7]. Along the same lines, the important effects of ligand-activated PPARγ in inhibiting the production of the VEGF in tumor endothelial cells should be further explored to counteract breast cancer angiogenesis [98]. Strategies aimed at eradicating BCSCs are also being examined for breast cancer treatment. In particular, since it has been largely reported that dysregulated Hedgehog, Notch and Wnt signaling pathways in BCSCs lead to breast tumor resistance, recurrence and metastasis, different drugs targeting these pathways have been developed and have reached clinical studies for breast cancer patients [116]. Interestingly, the ability of ligand-activated PPARγ to disrupt the BCSC niche has been described [38]. Thus, PPARγ agonists may represent potential agents for the BCSC-target therapy in breast cancer. Collectively, as summarized in Table 2, these data suggest that PPARγ could be a good target in the breast TME and its activation by natural and synthetic ligands may educate cells within TME generating an “unsupportive” milieu for breast tumor progression.

## 7. Conclusions

**Figure 1:**
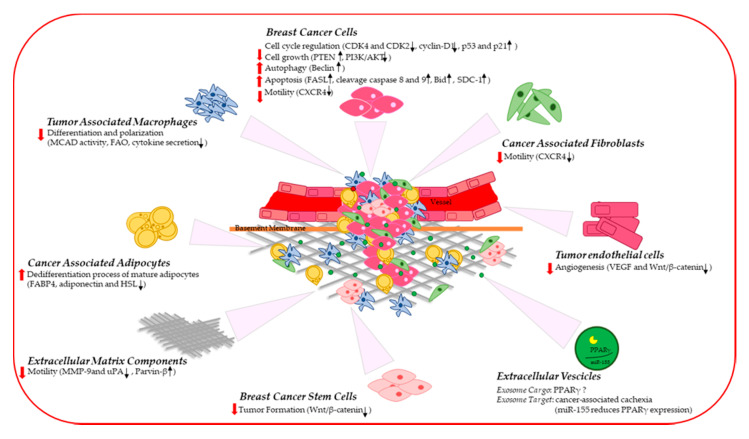
A schematic illustration showing the potential tumor suppressor role exerted by PPARγ in the breast cancer microenvironment. CDK: cyclin-dependent kinase; PTEN: phosphatase and tensin homolog on chromosome ten; PI3K/AKT: phosphatidylinositol 3-kinase/protein kinase B; FASL: FAS Ligand; SDC-1: syndecan-1; CXCR4: C-X-C chemokine receptor type 4; MCAD: medium-chain acyl-CoA dehydrogenase; FAO: fatty acid oxidation; FABP4: fatty acid binding protein 4; HSL: hormone-sensitive lipase; VEGF: vascular endothelial growth factor; MMP9: Matrix metallopeptidase 9; uPA: urokinase plasminogen activator.

Breast TME is recognized to be a key player in cancer progression and a promising therapeutic target in breast carcinoma. In a niche composed of an epithelial/stromal cellular part and ECM components, TME is a complex network of signaling and distinct tissue properties. The reciprocal cell–cell/ECM interaction and the ability of tumor cancer cells to force stromal cells to acquire malignant phenotypes contribute to promote breast cancer development and invasion. Disrupting cancer cell interplay may represent an effective therapeutic strategy to fight breast cancer. In this context, natural and synthetic PPARγ agonists have been proven to exert potent modulatory effects in different cell types, extending the repertoire of potential cellular target of this tumor suppressor. Indeed, PPARγ activation in the epithelial breast cancer cells results in a reduced cell growth and motility as well as an increased autophagy and apoptosis. Furthermore, ligand-activated PPARγ in the surrounding stromal components creates a milieu that hinders breast tumor progression (Figure 1). Unraveling the precise role of PPARγ in the complex tissue response in cancer could be paramount for a rational design of new therapy schemes that take advantage of the potent antitumor action of PPARγ agonists targeting both epithelial and stromal cells within a breast tumor microenvironment.

## Figures and Tables

**Table 1:** ‘In vitro’ and ‘in vivo’ studies showing the effects of proliferator-activated receptor γ (PPARγ) activation in breast cancer.

Cell Lines Animal Model	Effects	References
**21PT (HER2+)**	Terminal differentiation	[11]
**MCF-7 (ER+/PR+)**	Cell cycle arrest	[15,16,17]
**MCF-7**	Growth inhibition	[18,19]
**MCF-7 Bearing-Nude Mice**	Growth inhibition	[19]
	Apoptosis	[28]
**MDA-MB-231 (ER-/PR-/HER2-), MCF-7, SKBR3 (HER2+)**	Autophagy	[8,21,22]
**MCF-7, T47D (ER+/PR+), MDA-MB-231, MDA-MB-468 (ER-/PR-/HER2-), BT-20 (ER-/PR-/HER2-), SKBR3**	Apoptosis	[8,23,24,25,26,28,29,30,31]
**MDA-MB-231, MCF-7**	Migration inhibition	[7]

HER2: human epidermal growth factor receptor 2; ER: estrogen receptor; PR: progesterone receptor.

**Table 2:** Microenvironment components and the therapeutic potential of PPARγ ligands.

Target	Mechanistic	PPARγ Ligand	Evidence
TAMs	TAM reprogramming	DHEA, DHA-5HT	[9]
CAFs	CXCR4/SDF-1 signaling CAF reprogramming	Rosiglitazone Pioglitazone	[7,38,115]
TECs	VEGF secretion	Rosiglitazone, 15d-PGJ2, Pioglitazone	[94,98]
BCSCs	Notch signaling	Pioglitazone	[38]
ECM protein	MMP secretion	Pioglitazone, Rosiglitazone, 15d-PGJ2, GW7845, DHA, Troglitazone	[6,102,103]

TAMs: tumor-associated macrophages; CAFs: cancer-associated fibroblasts; TECs: tumor endothelial cells; BCSCs: breast cancer stem cells; ECM: extracellular matrix; CXCR4: C-X-C chemokine receptor type 4; SDF-1: stromal cell-derived factor-1; VEGF: vascular endothelial growth factor; MMP: matrix metallopeptidase; DHEA: docosahexaenoyl ethanolamine; DHA-5HT: docosahexaenoyl serotonin; 15d-PGJ2: 15-deoxy-Δ12,14-prostaglandin J2; DHA: docosahexaenoic acid.

## Abbreviations

AKT	protein kinase B
BCSCs	breast cancer stem cells
CAAs	cancer-associated adipocytes
CAFs	carcinoma-associated fibroblasts
CDKs	cyclin-dependent kinases
CLS	crown-like structures
CXCR4	C-X-C chemokine receptor type 4
DHA	docosahexaenoic acid
DHA-5-HT	*N*-docosahexaenoyl serotonin
DHEA	*N*-docosahexaenoyl ethanolamine
ECM	extracellular matrix
EMT	epithelial–mesenchymal transition
EPA	eicosapentaenoic acid
ER	estrogen receptor
ERBB2/HER2	Erb-B2 Receptor Tyrosine Kinase 2/Human Epidermal Growth Factor Receptor 2
EVs	extracellular vesicles
FABP4	fatty acid binding protein 4
FSP-1	fibroblast-specific protein-1
HIF1	hypoxia-inducible factor 1
HSL	hormone sensitive lipase
ILs	Interleukins
ILKs	integrin-linked kinases
LOX	lysyl oxidase
MCAD	medium-chain acyl-CoA dehydrogenase
MMPs	matrix metalloproteinases
PAI-1	plasminogen activator inhibitor type-1
15d-PGJ2	15-deoxy-Δ12,14-prostaglandin J2
PI3K	phosphatidylinositol 3-kinase
PTEN	phosphatase and tensin homolog on chromosome ten
PUFAs	polyunsaturated fatty acids
PPAR	peroxisome proliferator-activated receptor
PPRE	PPAR response element
ROS	reactive oxygen species
RXR	retinoid X receptor
SDC-1	syndecan-1
SDF-1	stromal cell-derived factor-1
SMA	smooth muscle actin
SMRT	silencing mediator of retinoid and thyroid hormone receptor
TAMs	tumor-associated macrophages
TIMP-1	metallopeptidase inhibitor 1
TGF	transforming growth factor
TNBC	triple negative breast cancer
TNF	tumor necrosis factor
TZDs	thiazolidinediones
TME	tumor microenvironment
TNF	tumor necrosis factor
uPA	urokinase plasminogen activator
VEGF	vascular endothelial growth factor
